# SNP genotyping elucidates the genetic diversity of *Magna Graecia* grapevine germplasm and its historical origin and dissemination

**DOI:** 10.1186/s12870-018-1576-y

**Published:** 2019-01-06

**Authors:** Gabriella De Lorenzis, Francesco Mercati, Carlo Bergamini, Maria Francesca Cardone, Antonio Lupini, Antonio Mauceri, Angelo Raffaele Caputo, Loredana Abbate, Maria Gabriella Barbagallo, Donato Antonacci, Francesco Sunseri, Lucio Brancadoro

**Affiliations:** 1 Dipartimento di Scienze Agrarie ed Ambientali, via Celoria 2, 20133 Milan, Italy; 2 Istituto di Bioscienze e Biorisorse CNR, Corso Calatafimi 414, 90120 Palermo, Italy; 3 Consiglio per la ricerca in agricoltura e l’analisi dell’economia agraria, Centro di ricerca Viticoltura ed Enologia, CREA-VE, via Casamassima 148, 70010 Turi, Bari Italy; 4 Dipartimento AGRARIA, località Feo di Vito snc, 89121 Reggio Calabria, Italy; 5 Dipartimento di Scienze Agrarie, Alimentari e Forestali, viale delle Scienze 11, 90128 Palermo, Italy

**Keywords:** SNP, Molecular markers, Genetic diversity, Secondary center of domestication, Parentage

## Abstract

**Background:**

*Magna Graecia* is the ancient name for the modern geopolitical region of South Italy extensively populated by Greek colonizers, shown by archeological and historical evidence to be the oldest wine growing region of Italy, crucial for the spread of specialized viticulture around Mediterranean shores. Here, the genetic diversity of *Magna Graecia* grape germplasm was assessed and its role in grapevine propagation around the Mediterranean basin was underlined.

**Results:**

A large collection of grapevines from *Magna Graecia* was compared with germplasm from Georgia to the Iberian Peninsula using the 18 K SNP array. A high level of genetic diversity of the analyzed germplasm was determined; clustering, structure analysis and DAPC (Discriminant Analysis of Principal Components) highlighted the genetic relationships among genotypes from South Italy and the Eastern Mediterranean (Greece). Gene flow from east (Georgia) to west (Iberian Peninsula) was identified throughout the large number of detected admixed samples. Pedigree analysis showed a complex and well-structured network of first degree relationships, where the cultivars from *Magna Graecia* were mainly involved.

**Conclusions:**

This study provided evidence that *Magna Graecia* germplasm was shaped by historical events that occurred in the area due to the robust link between South Italian and Greek genotypes, as well as, by the availability of different thermal resources for cultivars growing in such different winegrowing areas. The uniqueness of this ampelographic platform was mainly an outcome of complex natural or human-driven crosses involving elite cultivars.

**Electronic supplementary material:**

The online version of this article (10.1186/s12870-018-1576-y) contains supplementary material, which is available to authorized users.

## Background

Grapevine (*Vitis vinifera L. subsp*. *sativa*) is one of the most important economic fruit species in the modern world; of West Asiatic origin, it is cultivated in a wide area from Trans-Caucasus to Western Europe and around the Mediterranean Basin [1]. The wild form (subsp. *sylvestris*) is suggested to have first appeared about 65 million years ago and its domestication was closely related to winemaking [2–4]. This process occurred about 8000 years ago and took place in the Caucasus, in an area located between the eastern coast of the Black Sea and the southern coast of the Caspian Sea [2, 5, 6]. From there, domesticated grapevines spread to south-eastern regions of the Mediterranean. During the second half of 5rd millennium BC grapevine appeared in Southern Greece and then moved to the southern Balkans, Central and Western Europe throughout South Italy [7, 8].

Among European countries, Italy is one of the major wine producers. As stated in the OIV Statistical Report on World Vitiviniculture, the area under vines cultivation in Italy reached 690,000 ha in 2016, with 7.9 million tons of grape production for both wine and table grape markets and 50.9 million hectoliters of wine (http://www.oiv.int/en/). In the National Register of Italian Grapevines more than 650 varieties are included (http://catalogoviti.politicheagricole.it/catalogo.php), representing a slice of grapevine diversity, as assessed by genetic analysis of Italian germplasm collections, including both local varieties and the most widely known national varieties [9–15]. A high number of synonyms were detected among genotypes collected in different winegrowing areas [15–19]. However, the number of varieties included in the Italian ampelographic platform remains still large, because in the past sexual reproduction, by spontaneous crossing, was a practice as common as vegetative propagation, evident from the large number of parentage relationships identified among Italian cultivars [10, 14–16, 20–22]. The most ancient archeological evidence of viticulture in Italy dates back to the Epigravettian and Mesolithic periods, in Grotta del Romito (Cosenza, Calabria) and Grotta dell’Uzzo (Trapani, Sicilia), respectively, where seeds of wild grapevines have been identified [23, 24]. Sicily, together with the other regions of southern Italy (Calabria, Campania, Basilicata and Puglia), played a key role in the introduction of viticulture to Italy during Greek colonization and its next spread along the Italian Peninsula to reach southern France (Marseilles) and western Spain [25]. Based on cultural and historical references, the first varieties introduced to southern Italy were: i) “Biblia”, imported by the Siracusa king, Pollis d’Argo, from the north-eastern Aegean; ii) “Morghio”, introduced to the southern Bruzio (now known as Calabria); iii) “Lagaria”, introduced to the Metaponto area (Basilicata) [26]. The co-existence of these with the autochthonous cultivars would help to shape the complex ampelographic platform of the Italian Peninsula, as a region of intermixing and exchange of varieties, which resulted in an admixed genetic structure [12, 27]. These authors addressed a lack of structuring in the Italian grapevine germplasm related to historical events which occurred in this country (Greek colonization, Roman Empire, Spanish colonization) over the course of centuries, probably due to the continual exchange of grape plants inside and outside the Italian Peninsula.

According to archaeobotanical data, Italian grapevine domestication was mainly determined by local grape populations, sharing a genetic pool with varieties from the Hellenic world, where viticulture had already reached a high level of specialization [28, 29]. As a consequence, a secondary domestication center of grapevine arose, as a long-term process of hybridization and selection of suitable genotypes. This hypothesis is supported by the finding of seed remains exhibiting intermediate traits between the two subspecies *sylvestris* and *sativa*, on the islands of Filicudi and Salina (Sicily), referring to the Middle bronze age [29, 30]. The first evidence of a developed cultivation system, dating back to the Middle Bronze Age, was found at sites in Strepparo and Cento Moggie (Caserta, Campania), where grapevine branches were discovered [31].

The biodiversity of southern Italian grapevine germplasm has been widely investigated by SSR (Simple Sequence Repeat) to study genetic variability [9–11, 14, 32], identify homonymies/synonymies [10, 14, 16] and parental relationships [14–16, 19, 20]. Structure and genetic diversity of local germplasm from southern Italy matches its historical and geographical background, and many synonyms, homonyms and parent-offspring relationships have been confirmed. Moreover, the identification of cultivars, such as Sangiovese, showing several relationships with southern Italian germplasm strengthens the genetic complexity of this ampelographic platform [14–16, 20, 21, 32, 33].

Recently, programmes of sequencing and re-sequencing of the grape genome have generated a database including an extensive number of single nucleotide polymorphisms (SNP), useful for setting up different genotyping SNP-panels developed for both SNPlex™ and chip array strategies [13, 34–36]. An initial set of 10 K SNP loci obtained from 17 grape DNA samples (10 cultivated *V. vinifera* and 7 wild *Vitis* species) was developed by Myles et al. [36]. The GrapeReSeq Consortium developed the Vitis18KSNP chip array, holding 18,775 SNPs chosen from *V. vinifera* and *Vitis* spp. genotypes [35]. Finally, Marrano et al. [37] described a new set of 37 K SNP in a grapevine collection of cultivated and wild accessions through a novel protocol of restriction-site associated DNA sequencing.

The usefulness of SNP sets has been demonstrated despite the bi-allelic nature of this kind of molecular marker. They were proven adequate to investigate genetic variability, discriminating among *V. vinifera* populations, between wild and cultivated compartments of *V. vinifera* and among wild *Vitis* species [15, 33–35, 37–41]. Furthermore, SNP were able to infer genetic structure [13, 38] and to identify kinships [15, 34, 38, 42]. In addition, the advantages of SNP genotyping are: i) high reproducibility among laboratories, indeed normalization with reference varieties is not required; ii) locus availability, thousands or millions of SNP can be retrieved from genome sequences; iii) high-throughput, multiplexing hundreds or thousands of loci in one chip; iv) automatization, sample processing may be completely automated.

*Magna Graecia* is the ancient name of southern Italy (nowadays the regions of Basilicata, Calabria, Campania, Puglia and Sicilia) colonized by the Greeks during the eighth century BC. This area was influenced by Greek civilization, in terms of customs and traditions, such as language, religious rites and agriculture, including viticulture and its varieties [25]. Here, a large germplasm collection of grapevine accessions originating from *Magna Graecia* were genotyped by Vitis18kSNP chip array to identify synonymies/homonymies, to investigate genetic diversity, population structure and parentage. To highlight the east-to-west gene-flow between the primary (Caucasus region) and the secondary domestication centers, a SNP dataset with genotypes from Georgia up to the Iberian Peninsula were included in the analysis.

## Methods

### Plant material

**Fig. 1:**
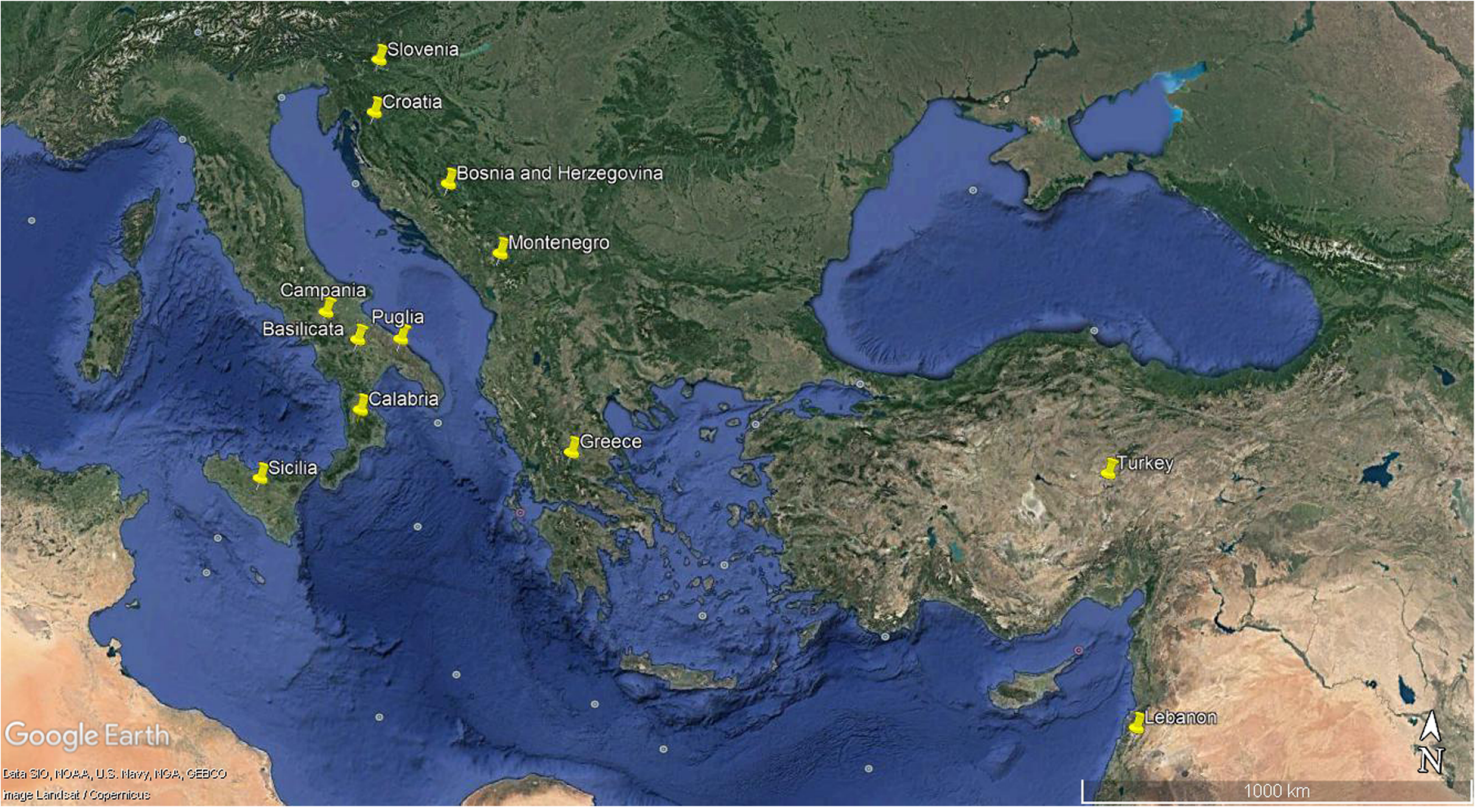
Map of the Countries and Italian regions where the 140 grapevine genotypes analyzed de novo come from. The image was created in Google Earth

A total of 140 grapevine accessions, originating from southern Italy, Greece and the Eastern Mediterranean Sea (Bosnia and Herzegovina, Croatia, Lebanon, Montenegro, Slovenia, Turkey) were genotyped by Vitis18kSNP chip array (Table 1; Fig. 1). The Italian samples were from five regions suited to viticulture from ancient times: Basilicata, Calabria, Campania, Puglia and Sicilia. To this set of samples, SNP profiles of 10 and 32 cultivars from Sicilia and Calabria, respectively, were included [15, 38], as well as SNP profile of Aglianico [41]. Aleatico, Moscato bianco, Pinot noir and Sangiovese profiles [38] were finally included in the dataset as reference varieties. A detailed list of plant material is reported in Additional file 1a, including passport data of accessions and SSR-molecular data at 9 loci (VrZag62, VrZag79, VVMD5, VVMD7, VVMD25, VVMD27, VVMD28, VVMD32, VVS2; [43–46]) already published or coming from our private databases. This sample set, encompassing 187 genotypes described above, is named sample set #1 from here on.

**Table 1:** Number of grapevine accessions genotyped de novo by 18 K SNP array and arranged based on their geographical origin

Populations	Number of genotypes
South Italy	111
Basilicata	15
Calabria	4
Campania	42
Puglia	25
Sicilia	25
Eastern Mediterranean Sea^a^	29
Total	140

^a^Genotypes originated in Bosnia and Herzegovina (4), Croatia (1), Greece (20), Lebanon (1), Montenegro (1), Slovenia (1) and Turkey (1)

To investigate the role of *Magna Grecia* germplasm into the domestication and dissemination process of grapevine, our sample set (#1) was added with SNP profiles already available. This second sample set (#2) included genotypes from sample set #1, SNP profile of 42 varieties coming from Georgia already described in De Lorenzis et al. [39] and a subset of 478 SNP profiles of cultivars coming from Georgia, Turkey, Cyprus, Greece, Lebanon, Balkans, Italy, France and Iberian Peninsula [22] (Additional file 1b). This second sample set, encompassing 709 genotypes described above, is named sample set #2 from here on.

### DNA extraction and genotyping

Genomic DNA was extracted from 100 mg of young leaf tissue by the DNeasy™ Plant Mini Kit (Qiagen - Hilden, Germany), following the manufacture’s instructions. The quality (260/230 and 260/280 ratios) and quantity of DNA extracted were checked using the NanoDrop Spectrophotometer (Thermo Fisher Scientific, Waltham, MA) and the Quant-iT dsDNA HS assay kit for Qubit 3.0 Fluorometer (Thermo Fisher Scientific), respectively.

The Vitis18kSNP array (Illumina Inc., San Diego, California), containing 18,071 SNPs, were used to genotype the 195 samples. The amplifications were performed on 200 ng of genomic DNA by the laboratory of Fondazione Edmund Much (San Michele all’Adige, Trento, Italy). SNP calls were scored with Genotyping Module 1.9.4 of the GenomeStudio Data Analysis V2011.1 software (Illumina Inc.). SNP loci showing call quality values (p50GC) lower than 0.54 were removed from the final dataset, as well as loci having GenTrain (GT) score values lower than 0.6. [39] and those with a percentage of missing data higher than 20% and minor allele frequency (MAF) lower than 0.05.

### Data analysis

#### Sample set #1

On the first sample set (#1), genetic distances and variability were assessed. The genetic variability was estimated by observed heterozygosity (H_o_), expected heterozygosity (H_e_) [47], the minor allele frequency (MAF), calculated by PEAS 1.0 software [48], and inbreeding coefficient (F), determined by R 3.4 software [49]. The genetic distances between genotypes was calculated by *poppr* [50] package implemented in R. The distance matrix was set up on Nei’s distances [51] and Unweighted Pair Group Method with Arithmetic Mean (UPGMA) algorithm was used for clustering. The circular dendrogram was plotted by using MEGA 5.0 software [52].

#### Sample set #2

On sample set #2, H_e_, H_o_, MAF and F indexes and cluster analysis were performed as described for sample set #1. The dendrogram was displayed as topology in MEGA 5.0 software [52], where the relationships among genotypes were viewed ignoring the branch lengths.

The genetic structure was investigated in more detail with a Bayesian model-based clustering algorithm implemented in *tess3* R package [53]. Based on SNP profiles, individuals were assigned to K populations, estimating the membership proportion for each genotype at each K. The program was run for ancestral population numbers ranging from K = 1 to K = 10. The algorithm was repeated 10 times for each K value, lambda value for the spatial regularization parameter was 1, the method chosen was “projected.ls” (an alternating projected least squares algorithm), with a maximum iterations number of the optimization algorithm up to 200 and a tolerance (value corresponding to the stopping criteria of the optimization algorithm) of 1e-05. The most likely K value was estimated inspecting the cross-validation curve and the results (membership proportion for each genotype at each K) were interpolated on a geographic map.

A Discriminant Analysis of Principal Components (DAPC; [54]) was performed to identify genetic clusters using the package *adegenet* of R software [55]. The maximum number of clusters was set to 10 and the number of axes considered in the Principal Component Analysis (PCA) was set to 60. The results were viewed as two-dimension scatter plot. H_e_ values were calculated and the genetic differentiation among DAPC clusters was validated by pairwise Nei’s standard genetic distance [52, 56] and pairwise Fst analysis [57], performed in R, using *nei.dist* function of *Poppr* package [50] and *pp.fst* function of *HierFstat* package [58], respectively. A Mantel test for Isolation-by distance (IBD) relationship was performed using the R software package *adegenet* [55] to calculate significant correlation among matrices of genetic (Nei’s and Fst) and geographical distances among the populations. Four-population test (A,B; C,D) implemented in TreeMix software [59] was used to test the gene flow among the clusters identified by DAPC. Standard errors for f-statistics were calculated in blocks of 500 SNPs (i.e. -K 500). A significant non-zero *Z*-score indicated gene flow between A,B and C,D. Higher values showed evidence of gene flow in the tree.

In order to infer relationships among non-redundant individuals, the identity-by-descent (IBD) index was calculated by PLINK 1.07 software [60]. IBD index counts for the probability that two genotypes are descended from single ancestral genotype and not identical by chance. MAF value was set at 0.1, while r^2^ of linkage disequilibrium value at 0.05. The relationships among pair of genotypes were assigned taking into account four parameters: Z0 (probability of sharing 0 IBD allele identical-by-descent), Z1 (probability to share 1 IBD allele), Z2 (probability to share 2 IBD alleles), and PI-HAT [(the relatedness measure measured as PI-HAT = P (IBD = 2) + 0.5 × P (IBD = 1)). A first-degree relationship (parent-offspring) was assigned to those pair of genotypes showing Z0 and Z2 values similar to 0, Z1 similar to 1 and PI-HAT to 0.5, a second-degree relationship when Z0 and Z2 showed values similar to 0.25, Z1 and PI-HAT to 0.5. An overview of first- and second-degree relationships among samples was displayed by circular visualization, obtained by Circlize R package [61]. The network of first degree relationships was obtained by R Network package [62].

## Results

### Genetic diversity of *Magna Graecia* germplasm

The genetic diversity among southern Italian grapevine accessions and those coming from Eastern Mediterranean Sea (Bosnia and Herzegovina, Croatia, Greece, Lebanon, Montenegro, Slovenia, Turkey), the so called sample set #1, were investigated by a high-throughput genotyping system based on SNP chip array, the Vitis18kSNP array. The final dataset counted 11,023 loci (Additional file 2) after the removal of: i) 73 loci (less than 1%) that did not amplify overall the genotypes; ii) 4616 loci (25%) that showed GT values lower than 0.6; iii) 1937 monomorphic loci (12% of dataset after GT filtration); iv) 495 loci (4% of the resultant dataset) with a MAF value lower than 0.05. All loci exhibited p50GC values higher than 0.54, while after inspection no genotypes with a percentage of non-calling loci higher than 20% were detected. H_o_ values were lower than the expected values from total germplasm within the sample set #1, ranging from 0.2873 (eastern Mediterranean Sea population) to 0.3060 (Italian population). Total H_e_ value was 0.3179 and the range varied from 0.3017 (Georgia) to 0.3471 (South Italy, Calabria) (Table 2). The overall value of MAF was 0.2206 (Table 2) and 1102 out of 11,023 SNP loci (about 10%) showed a MAF value lower than 0.100. The MAF values, similar among the different populations, ranged from 0.2176 (Greece) to 0.2270 (South Italy, Calabria) (Table 2). The values of F index (inbreeding coefficient) ranged from 0.1276 (Greece) to 0.1906 (South Italy, Calabria), with a value overall the population equal to 0.1496.

**Table 2:** Summary of genetic variation statistics at 18 K SNP loci on sample set #1

Populations	H_o_^a^	H_e_^b^	MAF^c^	F^d^
South Italy	0.3060	0.3251	0.2246	0.1654
Basilicata	0.3066	0.3280	0.2240	0.1605
Calabria	0.3059	0.3471	0.2270	0.1906
Campania	0.3099	0.3175	0.2268	0.1564
Puglia	0.3009	0.3173	0.2207	0.1378
Sicilia	0.3068	0.3154	0.2247	0.1425
Eastern Mediterranean Sea	0.2873	0.3124	0.2125	0.1321
Greece	0.2958	0.3037	0.2176	0.1276
Total	0.3001	0.3179	0.2206	0.1496

^a^Observed heterozygosity; ^b^Expected heterozygosity; ^c^Minor allele frequency; ^d^Inbreeding coefficient

A UPGMA dendrogram was built to investigate the genetic relationships among the genotypes from the 18 K SNP matrix data (Additional file 3). The range of similarity varied from 100 to about 83%. A total of 158 unique genotypes were detected, with the higher number of synonymies identified in two Italian regions, Calabria and Basilicata. Other synonymies were also noticed between genotypes from different Italian regions and from Italy and Eastern Mediterranean Sea (Kratosija and Primitivo) samples, as well. The synonymies identified among the *Magna Graecia* germplasm are listed in the Additional file 4. Among the Greek genotypes, Moschofilero and Mavrodaphni showed the same SNP profile. Cluster analysis was not able to define groups based on the geographic origin. Indeed, samples coming from different Italian regions were clustered together. Nevertheless, the Greek genotypes were clustered in a group including the most part of Eastern Mediterranean Sea samples. Pinot noir, Teran and some Italian genotypes clustered as the most distant genotypes, as outgroups.

### Genetic diversity of Mediterranean Basin germplasm

In order to compare the genetic relationship between *Magna Grecia* genotypes (included in the sample set #1) with that originating from neighboring winegrowing areas, from Georgia to the Iberian Peninsula, a second dataset called sample set #2 was built, including already available data from De Lorenzis et al. [39] and Laucou et al. [22]. This last set accounted for a total of 709 profiles at 7396 SNPs. Nineteen out of 709 genotypes were duplicated. The duplicated genotypes showed the same SNP profile at 99%. The inconsistencies were due to missing values. H_e_, H_o_, MAF and F indexes were measured, subdividing samples based on geographic area and historical influences in, from east to west, Georgia, eastern Mediterranean Sea Countries (Turkey, Greece, Cyprus, Lebanon and Balkans), South Italy, North Italy, France and Iberian Peninsula (Spain and Portugal); the values per each population are reported in Table 3. Eastern Mediterranean Sea and North Italy showed, respectively, the lowest and the highest values for both H_o_ (0.2819 and 0.3262) and H_e_ (0.3067 and 0.3406). Georgian population showed the lowest MAF value (0.2157), while North Italy population the highest (0.2368). About F index, the lowest value was detected for Georgia (0.1044) and the highest for South Italy (0.1830).

**Table 3:** Summary of genetic variation statistics at 18 K SNP loci on sample set #2

Populations	H_o_^a^	H_e_^b^	MAF^c^	F^d^
Georgia	0.3082	0.3107	0.2157	0.1044
Eastern Mediterranean Sea^e^	0.2819	0.3067	0.2223	0.1184
South Italy	0.3191	0.3392	0.2330	0.1830
North Italy	0.3262	0.3406	0.2368	0.1611
France	0.3205	0.3393	0.2332	0.1715
Iberian Peninsula^f^	0.3111	0.3275	0.2263	0.1502
Total	0.3112	0.3273	0.2279	0.1481

^a^Observed heterozygosity; ^b^Expected heterozygosity; ^c^Minor allele frequency; ^d^Inbreeding coefficient, ^e^Turkey, Greece, Cyprus, Lebanon and Balkans; ^f^Spain and Portugal

The genetic relationships among genotypes were detected from a SNP data matrix of 709 genotypes by using Nei’s distances (Nei 1972) and UPGMA algorithm (Additional file 5). The genotypes showed similarity values ranging from 88 to 100%. Within samples from South Italy and compared to genotypes from other populations, novel synonymies were not detected in addition to those already described above for “Genetic diversity of *Magna Graecia* germplasm”. Here three main clusters were highlighted, the largest included samples from Eastern Mediterranean Sea, South Italy and Iberian Peninsula (C1). The second included only samples from Georgia (C2), while the third cluster was mainly comprised of samples from North Italy and France (C3) (Additional file 5). Furthermore, a clear discrimination among genotypes coming from Eastern Mediterranean Sea, South Italy and Iberian Peninsula were observable in cluster C1, while 25% of Iberian Peninsula accessions were grouped in C3, together with the samples from France and North Italy.

### Population structure of *Magna Graecia* and Mediterranean Basin germplasm

**Fig. 2:**
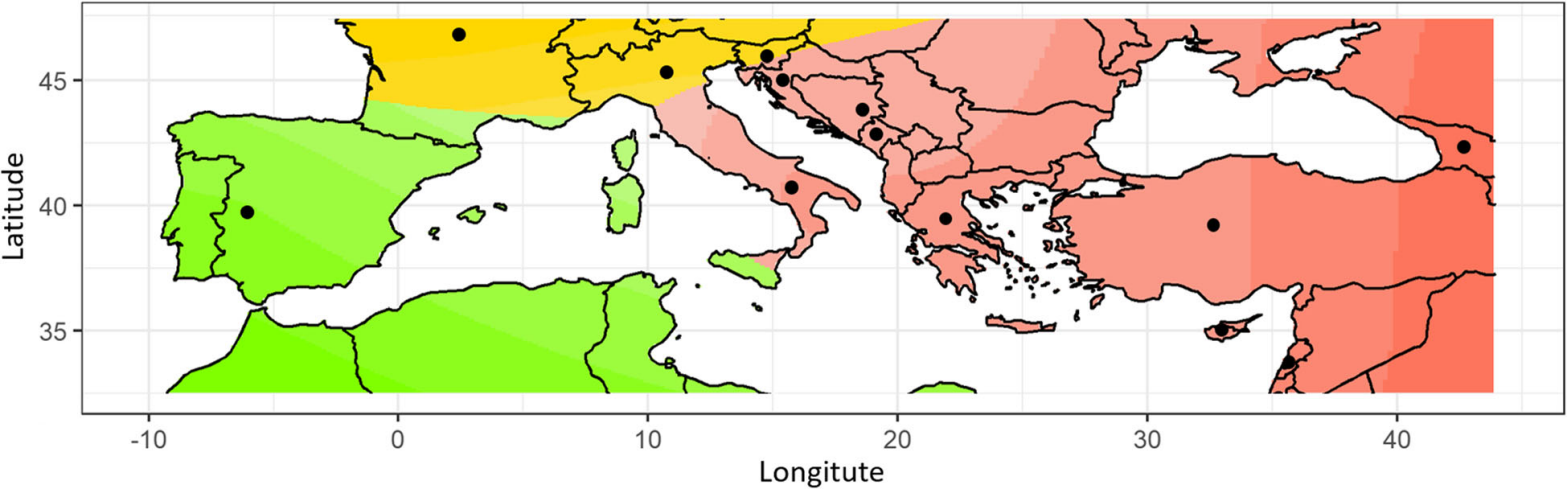
Geographic map of structure ancestry coefficients. Results of structure analysis performed on 709 grapevine accessions of sample set #2 (coming from Georgia, Turkey, Greece, Cyprus, Lebanon, Balkans, Italy, France and Iberian Peninsula) based on 18 K SNP profiles. The most likely number of ancestral groups was three. Genotypes from North and South Italy were split in two populations. Dots indicate the countries where the genotypes originated. The higher the colour shade the higher the percentage of membership

The likely number of ancestral genetic groups (K) within the germplasm from sample set #2 was investigated by *tess3* R package. The algorithm for cross-validation curve revealed K = 3. Fifty-nine out of 709 genotypes (around 8%) showed a percentage of membership > 80% (Additional file 6). All these structured genotypes were from Georgia. The ancestry coefficients calculated by *tess3* were spatially interpolated into a map (Fig. 2). Although the high number of admixed genotypes, the spatial interpolation of ancestry proportions inferred assigned genotypes from Georgia, Eastern Mediterranean Sea Countries and South Italy to the same cluster. In the second cluster, samples belonging to North Italy and France were mainly included, while the third one is limited Iberian Peninsula samples.

**Fig. 3:**
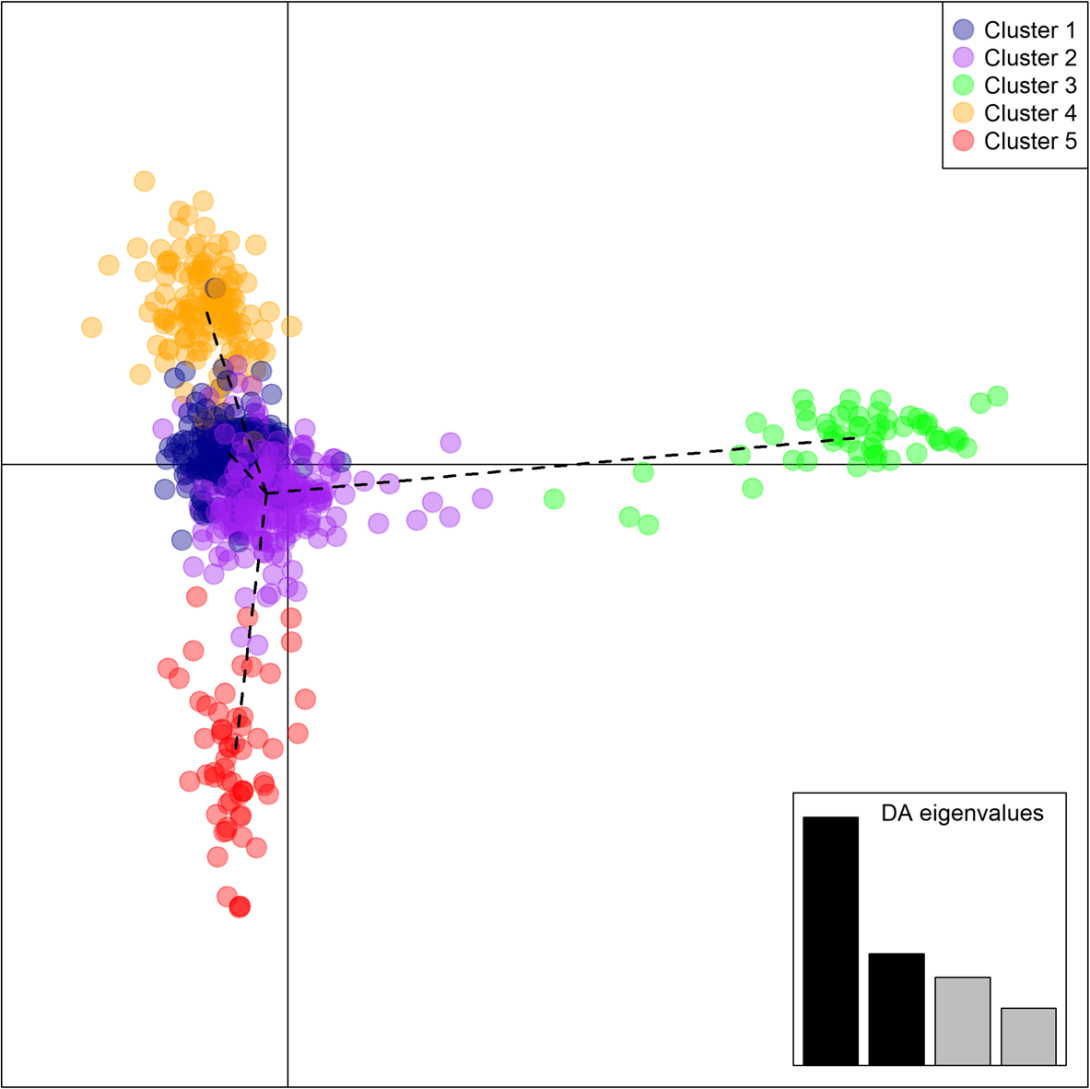
Two-dimension DAPC (Discriminant Analysis of Principal Component) scatter plot. Results of DAPC performed on 709 grapevine accessions of sample set #2 (coming from Georgia, Turkey, Greece, Cyprus, Lebanon, Balkans, Italy, France and Iberian Peninsula) based on 18 K SNP profiles. Genotypes were grouped in five clusters. Black lines represent a minimum-spanning tree based on the squared distances between five clusters identified. Cluster 1: France and North Italy; Cluster 2: Eastern Mediterranean Sea Countries and South Italy; Cluster 3: Georgia; Cluster 4: Iberian Peninsula; Cluster 5: South Italy

DAPC identified five clusters: Cluster 1 included mainly French genotypes (78%); Cluster 2 was the most assorted cluster, grouping together mainly samples from eastern Mediterranean Sea countries (97%) and South Italy (59%), together with Georgian, French, North Italy and Iberian Peninsula genotypes; Cluster 3 comprised only Georgian samples (87%); Cluster 4, 65% of Iberian Peninsula accessions, while Cluster 5 comprised only samples coming from the South Italy (31%) (Fig. 3 and Additional file 7). The plot of the first two principal components distinguished Clusters 3, 4 and 5, while the differences between Clusters 1 and 2 were less clear (Fig. 3). The five genetic clusters were not geographically restricted, except for Cluster 5, confirming a high level of genetic admixture. The minimum-spanning tree between populations based on the squared distances demonstrated an equidistance between samples from Cluster 2 and those included in Cluster 3, 4 and 5. H_e_ values of DAPC ranged from 0.2716 (Cluster 5) to 0.3073 (Cluster 1) (Table 4). The pairwise Nei’s genetic distance ranged from 0.0036 (Cluster 2 vs. Cluster 4) to 0.100 (Cluster 3 vs. Cluster 4), while the Fst index showed values ranging from − 0.633 (Cluster 3 vs. Cluster 4) and − 0.209 (Cluster 2 vs. Cluster 4) (Additional file 8).

**Table 4:** Expected heterozygosity (H_e_) values of five clusters inferred by DAPC

DAPC clusters	H_e_
Cluster 1	0.3073
Cluster 2	0.2978
Cluster 3	0.3041
Cluster 4	0.2772
Cluster 5	0.2716

Cluster 1: France and North Italy; Cluster 2: Eastern Mediterranean Sea countries and South Italy; Cluster 3: Georgia; Cluster 4: Iberian Peninsula; Cluster 5: South Italy

The geographical patterning suggested by the DAPC analysis was verified by a Mantel test to detect the isolation-by distance. In both analyses, a correlation between genetic (Nei’s and Fst) and geographical distance matrices was found (Additional file 9), with *r* = 0.751 and − 0.789 for geographical distance matrix and Nei’s and Fst matrices, respectively.

The four population test *f*4 (A, B; C, D) tests whether population A and B vs. population D and C can be considered distinct clades in a population tree. The groups showing the highest non-zero Z-score values, suggesting gene flow, were Cluster 1 and 5 vs. Cluster 2 and 3 (Z-score = − 7.2797) and Cluster 1 and 4 vs. Cluster 2 and 3 (Z-score = − 9.2147) (Additional file 10).

### Parentage analysis of *Magna Graecia* germplasm

The first (PO, parent-offspring) and second-degree (grandparent–grandoffspring, halfsiblings or uncle–nephew) relationships were detected among 709 genotypes included in sample set #2 to define a proposed pedigree of southern Italian samples. Z0, Z1, Z2 and PI-HAT values for PO and second-degree relationships detected among Southern Italian samples were reported in Additional file 11. Up to 82 genotypes from South Italy shared almost one PO relationship, while 100 PO relationships were detected (Additional file 11, Additional file 12). Most relationships were identified between Southern Italian genotypes. Inter-population relationships (between South Italy genotypes and other populations) were also identified, such as between Puglia and Eastern Mediterranean Sea samples (Primitivo and Blatina, Pampanuto and Plavina), Calabria and Eastern Mediterranean Sea samples (Lacrima bianca and Ladikino), Campania and Iberian Peninsula (Gloria and Breval negro).

**Fig. 4:**
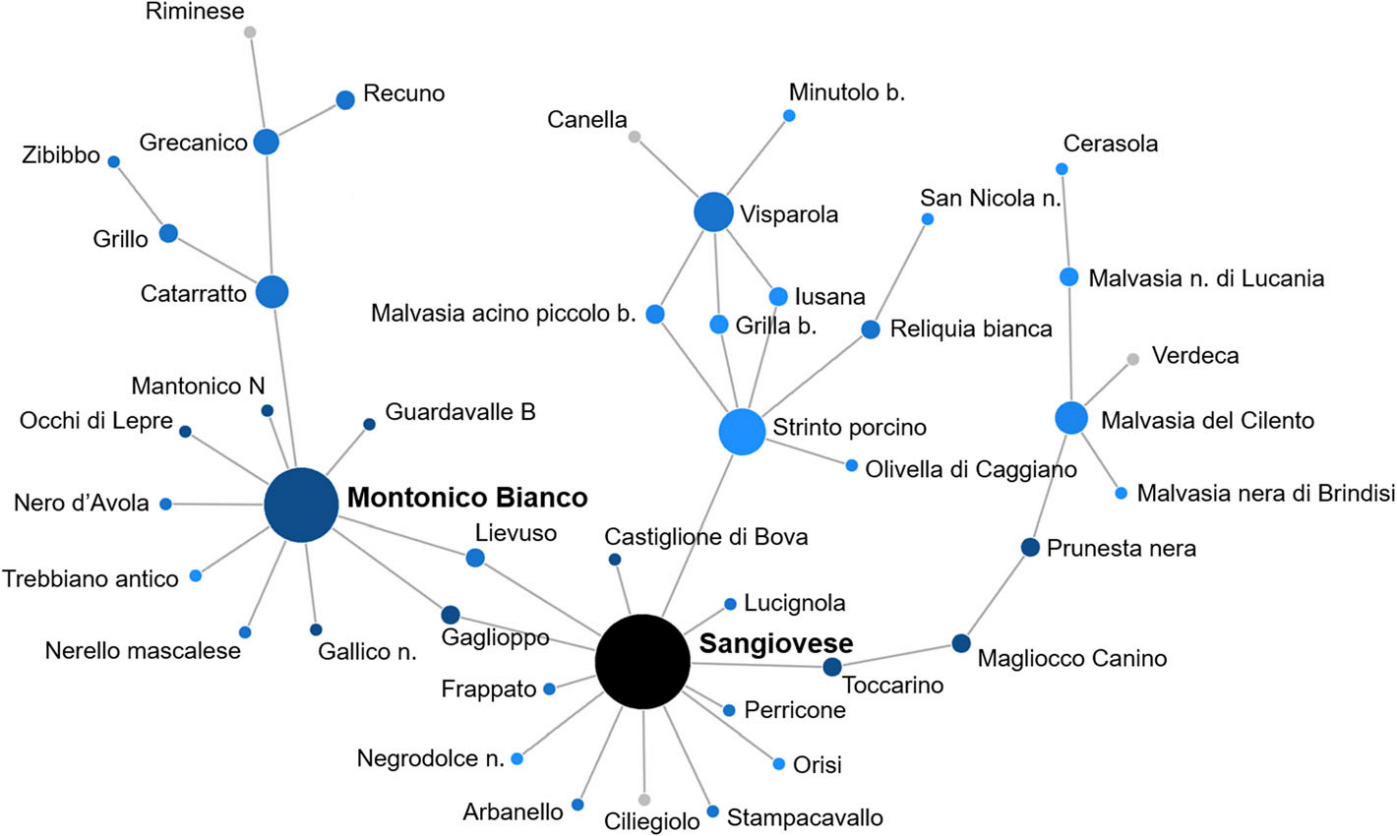
Network of first-degree relationships of Mantonico Bianco and Sangiovese. Vertices were colored based on the geographical origin of genotypes (Italy: blue scale based on sub-populations; genotypes from Laucou et al. [22]: grey; Reference: black) and their size was scaled based on the number of first-degree relationships of each genotype

The cultivars with the highest number of relationships were Sangiovese (13) and Mantonico Bianco (10) (Fig. 4, Additional file 11, Additional file 12). Sangiovese shared kinship relations with cultivars from four out of five Italian regions investigated: Basilicata (Stampacavallo, Strinto porcino), Calabria (Castiglione di Bova, Gaglioppo, Toccarino), Puglia (Negrodolce n.) and Sicily (Arbanello, Frappato, Lievuso, Lucignola, Orisi, Perricone). Further, Sangiovese showed PO relationships with Ciliegiolo [22]. Mantonico Bianco showed first degree relationships with genotypes from Basilicata (Trebbiano antico), Calabria (Gaglioppo, Gallico n., Guardavalle B, Occhi di Lepre, Mantonico N) and Sicily (Catarratto, Lievuso, Nerello mascalese and Nero d’Avola).

Other genotypes showing a well-defined pedigree-network were Bombino bianco (7 PO relationships) and Aglianico (6). Bombino bianco exhibited Z0, Z1, Z2 and PI-HAT values similar to PO relationship with Bombino n., Cococciola, Colatamurro, Impigno b., Montepulciano, Sanguinella b. and Uva di Troia (Additional file 11, Additional file 12). Aglianico showed PO relationships with Aglianico antico, Aglianicone, Aglianicone di Cardile, Cannamelo n., Rossa di Moico and Rossa di San Nicola (Additional file 11, Additional file 12).

Eight genotypes showed a second degree relationship, for a total of six relationships (Additional file 11, Additional file 12). These second degree relationships have been identified among Italian samples, and between Nerello cappuccio (Sicily) and Morate from Spain**.**

## Discussion

### *Magna Graecia* germplasm has a high genetic diversity

The 18,071 SNPs held in the Vitis18kSNP array were used to investigate the genetic diversity of grapevine germplasm native to *Magna Graecia*. Due to its centrality in the Mediterranean Basin and political and economic contacts with other Mediterranean countries, the genetic relationships between *Magna Graecia* germplasm and those from the neighboring countries and the primary grape domestication center [2] were assessed. To accomplish genetic characterization, two different datasets were built. The first one included samples from *Magna Graecia*, Greece and some samples from the Balkans, called sample set #1 (Table 1 and Additional file 1a). The sample set #2 was built adding to the first SNP profiles of grapevines from Mediterranean area (from Georgia to Portugal) previously reported [22, 39] (Additional file 1b).

In the sample set #1, all the accessions were successfully genotyped, resulting 11,023 polymorphic SNPs (Additional file 2), after filtering loci that did not amplify overall the entire dataset, that did not reach the GT threshold value of 0.6, monomorphic and with a MAF value lower than 0.05. Even though the dataset was assorted, the number of monomorphic loci was quite high, reaching 12%. However, the number of loci that did not show amplification (1%) was reasonable taking into account that 25% of SNP loci included in the array were from *Vitis* genomes but not from *V. vinifera* [35]. This result is comparable with those previously reported [15, 38, 39]. H_o_ values (of approximately 0.3000) were similar to H_e_ values (0.3179 across populations), suggesting that the populations can be affected by frequent inbreeding events (Table 2). These data supported previous observations based on both different [13, 33, 34, 37] and identical sets of SNPs [15, 38, 39]. The Italian population and its subpopulations showed the highest H_e_ values, indicating a relatively high level of genetic variability. The distribution of MAF values (Table 2) and the percentage of loci showing MAF values lower than 0.05 (4%) was quite similar to those observed using different SNP panels [13, 34, 37]. The low F values (0.1496 across populations; Table 2) were justified by the high level of heterozygosity observed, as previously stated [3, 63].

After merging genotypes from Georgia and Mediterranean Basin Countries [22, 39] with sample set #1, the resulting sample set #2 counted 709 samples genotyped at 7396 SNP loci. Duplicated genotypes showed 99% of homology in SNP profile, demonstrating the high reproducibility of this genotyping tool [38], and the ease of merging large datasets from different scientific reports [22]. Likewise, sample set #2 showed high values of heterozygosity (total H_o_ = 0.3112, total H_e_ = 0.3273) and H_o_ values higher than H_e_ values (Table 3). The eastern Mediterranean Sea population showed the lowest heterozygosity values for both datasets (Table 2 and Table 3), while the highest genetic diversity was detected for genotypes coming from North Italy (Table 3). MAF and F values were similar between the two datasets, Georgia showed the lowest values for both MAF and F indexes, while northern and southern Italian populations showed the highest MAF and F values, respectively (Table 3). The similarity of genetic variation statistics between the two datasets suggests that those parameters are not affected by population size and number of loci.

### The common genetic background of *Magna Graecia* germplasm

Cluster analysis on sample set #1 identified 22 cases of synonymies (Additional file 3, Additional file 4), most of which were detected among Italian samples. Some cultivars were identified in more than one region, such as Nerello mascalese (from Sicilia), also identified in Calabria and Campania. Zibibbo sampled in Campania and Sicilia, Primitivo in Campania and Montenegro (Kratosija), as already reported in other studies [10, 14, 15, 21, 64–67]. Many known synonymies were confirmed, while the remaining eight cases of synonymies were identified here for the first time (Additional file 4). The Moschofilero-Mavrodaphni synonymy was a clear misnaming, since they are supposedly different genotypes [68].

The clustering did not reflect the geographical origin of samples from South Italy, proving the common genetic background; even though some cultivars were clustered as the outgroup (Additional file 3). Moreover, the grouping of Italian and Greek samples together in the same clusters are in agreement with the historical events joining these two areas [25]. These results supported the hypothesis that during Greek colonization, Calabria and Sicilia played an important role for evaluating the potential of varieties coming from the Eastern Mediterranean Sea and their spreading firstly in South Italy and afterwards in Etruscan Italy (Central Italy) and France [25, 69]. During the assessment, the imported varieties could be crossed with both wild and domesticated local grapevines, overlapping with a domestication process of wild grapevines already under way at that time [70].

### The uniqueness of Georgian grapevine germplasm

The Caucasus is the place where grapevine domestication took place [71]. The genetic diversity of wild and cultivated grapevine germplasm from Georgia was extensively investigated by SSR and SNP molecular markers [14, 22, 27, 34, 72, 73]. The results demonstrated the uniqueness and originality of this germplasm due to distinctive traits compared to the other European samples, as well as the high level of heterozygosity. This uniqueness was confirmed by most of the statistics applied here. Cluster analysis and DAPC strongly separated Georgian samples from the others (Additional file 5, Fig. 3). This differentiation was also stated by pairwise Nei’s and Fst genetic distance values (Additional file 8). Indeed, pairwise analyses including Georgian genotypes showed the highest and lowest values for Nei’s and Fst genetic distance, respectively. Despite the high level of admixture, structure analysis identified three ancestral populations (Fig. 2). Georgian germplasm was grouped with the genotypes from the eastern Mediterranean Sea and South Italy, but appeared as unique population with well-structured genotypes, with a percentage of membership > 80% (Additional file 6). The same result was also highlighted by the spatial interpolation of the ancestry values (Fig. 2).

### The grapevine distribution route from Georgia to Iberian Peninsula through *Magna Graecia*

Domesticated grapevine spread gradually from the Caucasus westwards via Anatolia and Greece by different Peoples and from this area into the South Italy by the Greeks [71]. In Greece, the most ancient evidence of viticulture dates back to the 5th millennium BC [74, 75], while in Italy, the beginnings of grapevine cultivation would have started during the ninth century BC [76].

Myles et al. [34] suggested the hypothesis of an east-to-west flow of genetic resources from the primary domestication center. Here, this hypothesis was strongly confirmed by the spatial interpolated ancestry coefficient map, DAPC and cluster analysis (Fig. 2, Fig. 3, Additional file 5). Georgian germplasm is distinguishable from the others, appearing closely related mainly to eastern Mediterranean Sea and South Italian populations. Gene flow was also highlighted by four population test, indicating gene flow between French/North Italy and Iberian Peninsula genotypes (A,B; Cluster 1, Cluster 4) and South Italy/Eastern Mediterranean sea and Georgia genotypes (C,D; Cluster 2, Cluster 3) (Additional file 10). This result was confirmed by the high level of admixture detected by structure analysis (Additional file 6) and low Fst values (Additional file 8), indicating low population structuring and limited barriers to gene flow.

Grapevines were already found to have spread from *Magna Graecia* to Spain and France [71, 77]. Nevertheless, a relationship between South Italy and the Iberian Peninsula germplasm was only detected in cluster analysis (Additional file 5), suggesting a different genetic origin of this last population. Arroyo-Garcia et al. [78] suggested a secondary domestication center in Western Mediterranean regions (Iberian Peninsula, Central Europe and Northern Africa) based on chloroplastic DNA haplotype differences. This statement was partially confirmed by our analyses. Indeed, Iberian Peninsula germplasm was grouped aside from the French germplasm by structure analysis, DAPC and clustering (Fig. 2 and Fig. 3, Additional file 5). These results can be interpreted as the existence of different genetic backgrounds contributing to the genetic make-up of current grapevines of Iberian Peninsula and France. This different genetic background may possibly be related to different grapevine *sylvestris* populations. Wild populations of different genetic background were identified in Spain, resulting from disconnected glacial refugees in the Iberian Peninsula during the last Pleistocene glaciations [79]. Individuals from these wild populations contributed to the development of germplasm from Iberian Peninsula [73]. Conversely, Central European germplasm was reported to be affected by the admixture confluence of migration routes radiating from separate refugees during the postglacial era [80].

### Biodiversity of northern and southern Italian germplasm as a mirror of historical events

Structure population analysis applied to large grapevine collections revealed genetic groups defined mainly by subspecies (*sativa* or *sylvestris*), geographic origin and usage (wine or table grapes; [13, 27]). In this study, the main subdivision based on the geographical origin of samples was not totally captured. Focusing on whole Italian germplasm, an interesting clear discrimination among North and South Italy samples was observed. Structure, DAPC and cluster analysis grouped aside the genotypes from North and South Italy (Fig. 2, Fig. 3 and Additional file 5). Northern Italian genotypes mainly clustered with French germplasm while those from South Italy with Eastern Mediterranean Sea (clustering and DAPC) and Eastern Mediterranean Sea and Georgia (structure analysis).

In attempting to back-track the origin of this differentiation, this result can be explained by the different historical and demographical events occurred in the two areas. Italy was fragmented country until 1861 when the *Regno d’Italia* (Kingdom of Italy) was established, an united kingdom encompassing the entire Italian Peninsula. Apart the periods when the Italian territory was politically unified, such as under the domination of the Roman Empire or the Ostrogoths, different peoples have alternately occupied Italian territory over the course of the centuries. During classical age, Phoenicians and Greeks established settlements in the South Italy and the Celts inhabited the North Italy, while in the 8-9th century the Frankish Empire and Normans defeated North and South Italy, respectively, proclaiming the end of Italian political unity for the next 1300 years. Thus, rather than a division by geographic area, our data suggested a division by historical events.

Another aspect that might have been influenced the differentiation between northern and southern Italian germplasm is the different availability of thermal resources for cultivars growing in such different environments, characterized by different latitudes. Indeed, the thermal resources observed in the South Italy are similar to South-East Europe and the these observed in the North Italy are similar to North-East Europe [81].

### Secondary grapevine domestication centers

In invasive species, genetic diversity is expected to decrease from the origin of introduction to newly invaded areas [82]. *V. vinifera* subsp. *sativa* can be considered an invasive species, though the grapevine dissemination was mainly mediated by human migrations, taking into account the first center of domestication as the origin of introduction.

From east to west, H_e_ values decreased from Cluster 1 (the origin of introduction; H_e_ = 0.3041) to Cluster 2 (the first newly invading area; H_e_ = 0.2978). H_e_ increased to a maximum for Cluster 1 (0.3073), including samples from France and North Italy (Table 4). France and North Italy showed the highest H_e_ values when samples were arranged based on geographic origin (Table 3).

Where H_e_ values increase along the newly invaded areas, an event that maximized genetic diversity can be hypothesized. Domestication of a species from its wild relatives is an event that maximizes the genetic diversity of such species. Secondary domestication centers along the grapevine dissemination routes were proposed. These centers refer to places where spontaneous grapevines were of interest for local people [4] and are located in the southern Balkans and Aegean Region, South Italy [70], the western Mediterranean [78], Provence-Northern Italy and Central Europe. Nevertheless, this evidence is not enough to explain the high He values for French-Northern Italian genotypes.

Another aspect influencing the genetic diversity of a germplasm is the convergence of different populations in the hybrid zones. The hybrid zones are meeting areas of two populations as they expanded their ranges from separate glacial refuges. In Europe, three well-known hybrid zones were identified, in Western Central Europe along the French-German border, in the Alps and in Scandinavia [83]. Thus, the Central Europe is a melting pot, an admixture confluence of migration routes radiating from separate refuges. Postglacial range expansion, domestication of local wild individuals, conjunction of endemic varieties with highly diverse traits from distinct geographical regions migrating along the Balkan route and from the ancient Roman province of Pannonia [84], as well as socio-political events can help us to interpret the convergence of french-northern Italian grapevine germplasm.

### The complex pedigree of *Magna Graecia* germplasm

The inherited grapevine germplasm arose by spontaneous or man-made crosses among the cultivars and their vegetative propagation across the centuries from the initial domestication to the present day. Thus, cultivars are strictly related by parentage relationships each other resulting into a complex pedigree, as already described in previous reports [12, 34, 66]. The same complex pedigree was observed in our dataset, with 82 genotypes showing a parentage relationship within the dataset (Additional file 11, Additional file 12), confirming 35 previously described PO relationships and suggesting 65 new ones. These genotypes showed such a complex number of inter-relationships that DAPC grouped them as a separate group (Cluster 5) from the remaining genotypes of Southern Italy that clustered together with the Greek samples (Cluster 2) (Fig. 3).

Most relationships were detected among South Italy cultivars, supporting the hypothesis of an intense regional exchange of plant material and a complex natural or anthropogenic breeding. Nevertheless, relationships between genotypes from South Italy and those from eastern and western countries were also detected.

The cultivars showing the highest PO relationships were Sangiovese, Mantonico Bianco, Bombino bianco and Aglianico. The genetic diversity of grape was dramatically threatened when at the end of nineteenth century phylloxera (*Daktulosphaira vitifoliae*) reached Europe from North America and devastated European vineyards. The introduction of North America non-*vinifera* cultivars as rootstock safeguarded the future of European *V. vinifera*. At the same time, together with the ever more increasing practice of non-*vinifera* rootstocks the selection and vegetative propagation of elite cultivars occurred, resulting in the further reduction of grapevine genetic diversity [3]. At the Italian scale, this was confirmed by the identification of a high number of synonyms for the most important Italian cultivars [10–12]. Moreover, our results revealed that as the vegetative propagation of elite cultivars occurred so did the use of elite cultivars in grape breeding programs. These cultivars have shaped the grapevine ampelographic platform of the Southern Italy. The elite cultivars (such as Sangiovese and Aglianico) were varieties widespread in many important winegrowing areas or minor varieties but with large local interest (such as Mantonico Bianco and Bombino bianco). Similar results emerged when different progenies of Pinot (a worldwide cultivar) and Gouais blanc (a neglected cultivar) were identified [85].

The interest in looking for the origin and kingroup of Sangiovese, one of the most important Italian cultivars, is notable. Indeed, different pedigrees have been proposed [20, 86], as well as a large number of progenies and synonymies [12, 14–16, 32, 38]. The present study adds two new cultivars to the already long list of Sangiovese progenies. Toccarino and Strinto porcino, local cultivars from Calabria and Basilicata, respectively, attested and corroborated the strong relationship of Sangiovese with the southern Italian germplasm [12, 14–16, 20, 32, 38]. This result is strengthened by the clustering of Sangiovese within southern Italian samples (Additional file 5). Mantonico Bianco is less well known than Sangiovese, it is an ancient autochthonous cultivar from Calabria, mainly cultivated in the Locride area (nowadays the Province of Reggio Calabria) located on the Ionian Coast, one of the first provinces of *Magna Graecia*. The first evidence of Mantonico Bianco cultivation is dated back to 1600 [87]. Despite its limited spread, Mantonico Bianco showed PO relationships with cultivars from Calabria, Sicilia and Basilicata, already described in elsewhere [12, 14, 15, 19, 21, 66] and new cultivars (Gallico n., Occhi di Lepre, Nero d’Avola and Trebbiano antico).

Bombino bianco is a cultivar widespread in the South Italy, mainly in Puglia, Basilicata, Abruzzo, Lazio and Marche. It is the progenitor (together with Uva rosa antica) of Uva di Troia, Bombino nero and Impigno [88]. In the present study, four other siblings of Bombino bianco were identified: Cococciola, Colatamurro, Montepulciano and Sanguinella b.

Aglianico is an ancient grapevine cultivar mainly cultivated in Campania (biotypes Taburno and Taurasi) and Basilicata (biotype Vulture), and strongly related with the establishment of Greek colonies in Campania [89]. Here, Aglianico was identified as progenitor of several minor accessions from Basilicata and Campania. The progenitors of Aglianico are still unknown, but a putative shared parent between Aglianico and Dureza (a minor and neglected variety cultivated in the Rhône Alpes area [90]) was discovered. Nevertheless, PO relationships were not identified between Aglianico and French genotypes.

Another interesting cultivar was Gloria, sampled in Campania and showing a PO relationship with genotypes coming from Greece, Italy, France and Spain. This admixed progeny by geographic origin mirrors the shuffling and exchanges of cuttings that occurred over the centuries following migration routes in Europe and confirms the route from Greece to France and the Iberian Peninsula through South Italy.

The name Malvasia was used to identify a group of cultivars diverse for both genetic and phenotypic profiles, cultivated in many Mediterranean countries [91–93]. In the list of PO relationships detected among the sample set #2, a large number of relations including Malvasia genotypes were detected. The majority of these genotypes were not related each other, highlighting the heterogeneity of plants labelled as “Malvasia”.

## Conclusions

Archaeological and historical data suggested that grapevine primary domestication took place in the Caucasus and from there spread to South Italy *via* Greece, in at least two separate steps. In this study, an extensive genetic characterization of *Magna Graecia* grapevine germplasm, from Georgia to the Iberian Peninsula, was carried out by 18 K SNP loci. Based on genetic analysis, *Magna Graecia* germplasm showed a high level of heterozygosity and distinctive traits, such as a common genetic background and a complex pedigree. Nevertheless, a significant degree of gene flow was observed in agreement with historical and socio-eco-political events that occurred in the Mediterranean Basin. These results highlighted the central role of *Magna Graecia* in the spread of grapevine through western Europe, supporting the hypothesis of an intense exchange of plant material as well as a close relationship among southern Italian cultivars and genotypes from eastern and western countries. Moreover, the genetic diversity of *Magna Graecia* germplasm has been shaped by the historical events that occurred in this area and by the variability of atmospheric driving variables (such as temperature, solar radiation and humidity) that trigger selective pressures and determine productivity, quality and territorial specificity of agroforestry productions.

## Additional files

Additional file 1: **a** Passport data of 187 grapevine genotypes coming from Italy (Basilicata, Calabria, Campania, Puglia and Sicily), Eastern Europe (Bosnia and Herzegovina, Montenegro, Slovenia, Turkey) and Greece, the so-called sample set #1, and allele profiles at 9 SSR loci (when available). **b** List of genotypes added to the sample set #1 to obtain the sample set #2. The genotypes included belong to Georgia, Bosnia and Herzegovina, Cyprus, Spain, France, Greece, Italy, Portugal and Turkey (from De Lorenzis et al. [39] and Laucou et al. [22]). (XLSX 49 kb)

Additional file 2: SNP profiles of 187 grapevine accessions (sample set #1), genotyped at 18 K loci. Original dataset was filtered based on SNP call quality and GenTrain score: samples with low SNP call quality (p50GC < 0.54) were removed from the analysis and only SNPs with a GenTrain score higher than 0.6 were retained. “A”: homozygous for dominant allele; “B”: heterozygous for recessive allele; “?”: missing data. (XLSX 7910 kb)

Additional file 3: UPGMA dendrogram of 187 grapevine genotypes analyzed by 18 K SNP array. The samples were marked based on their geographical origin. South Italy: blue dots; Eastern Mediterranean Sea: red dots; Reference: black dots. (TIFF 1630 kb)

Additional file 4: List of synonymies identified among the *Magna Graecia* germplasm (sample set #1) analyzed by 18 K SNP array. (DOCX 13 kb)

Additional file 5: Topology of UPGMA dendrogram of 709 grapevine accessions genotyped with 18 K SNP array. Georgia: violet branch lines; Eastern Mediterranean Sea: red branch lines; South Italy: blue branch lines; North Italy: dark green branch lines; France: light green branch lines; Iberian Peninsula: cyan branch lines. C1, C2, C3: main clusters identified. (TIF 2662 kb)

Additional file 6: Ancestry values at K = 3 inferred by structure analysis on 709 grapevine accessions (sample set #2) coming from Georgia, Eastern Mediterranean Sea Countries (Turkey, Greece, Cyprus, Lebanon, Balkans), Italy (North and South), France and Iberian Peninsula (Spain and Portugal) genotyped at 18 K loci. (XLSX 56 kb)

Additional file 7: List of five cluster inferred by DAPC on 709 grapevine accessions of sample set #2 genotyped at 18 K loci. (XLSX 27 kb)

Additional file 8: Nei’s standard genetic distance (below the diagonal) and Fst index (above the diagonal) calculated on five clusters inferred by DAPC. (DOCX 12 kb)

Additional file 9: Graphical representation of correlations between genetic distances and geographical distance. A: Nei’s genetic distance; B: Fst genetic distance. Red: high correlation between genetic and geographical distances; Yellow: medium correlation between genetic and geographical distances; Blue: low correlation between genetic and geographical distances. Dots represent distance values between two populations detected by DAPC. (TIFF 1753 kb)

Additional file 10: Summary of four-population tests on five clusters inferred by DAPC. (DOCX 13 kb)

Additional file 11: Parent-offspring (PO) and second degree (2°) relationships identified for South Italy cultivars genotyped at 18 K SNP loci. Z0: probability to share 0 allele; Z1: probability to share 1 allele; Z2: probability to share 2 alleles; PI HAT: probability to be identical by descendent. (XLSX 18 kb)

Additional file 12: Circular representation of first (red links) degree relationships identified for South Italy cultivars genotyped at 18 K SNP loci. The samples are arranged based on their geographic origin. Italy: blue scale (based on sub-populations); Eastern Mediterranean Sea Countries: red; genotypes from Laucou et al. [22]: grey; Reference: black. (TIFF 19774 kb)

## Abbreviations

DAPC: Discriminant Analysis of Principal Components
F: Inbreeding coefficient
GT: GenTrain
He: Expected heterozygosity
Ho: Observed heterozygosity
IBD: Identity-by-descent
MAF: Minor allele frequency
p50GC: Call quality values
PCA: Principal Component Analysis
PI-HAT: The relatedness measure measured as PI-HAT = P (IBD = 2) + 0.5 × P (IBD = 1)
SNP: Single nucleotide polymorphism
SSR: Simple Sequence Repeat
UPGMA: Unweighted Pair Group Method with Arithmetic Mean
Z0: Probability of sharing 0 IBD allele identical-by-descent
Z1: Probability to share 1 IBD allele
Z2: Probability to share 2 IBD alleles
