# Correction to: SNP genotyping elucidates the genetic diversity of Magna Graecia grapevine germplasm and its historical origin and dissemination

**DOI:** 10.1186/s12870-020-02515-z

**Published:** 2020-07-02

**Authors:** Gabriella De Lorenzis, Francesco Mercati, Carlo Bergamini, Maria Francesca Cardone, Antonio Lupini, Antonio Mauceri, Angelo Raffaele Caputo, Loredana Abbate, Maria Gabriella Barbagallo, Donato Antonacci, Francesco Sunseri, Lucio Brancadoro

**Affiliations:** 1 Dipartimento di Scienze Agrarie ed Ambientali, via Celoria 2, 20133 Milan, Italy; 2 Istituto di Bioscienze e Biorisorse CNR, Corso Calatafimi 414, 90120 Palermo, Italy; 3 Consiglio per la ricerca in agricoltura e l’analisi dell’economia agraria, Centro di ricerca Viticoltura ed Enologia, CREA-VE, via Casamassima 148, 70010 Turi, Bari, Italy; 4 Dipartimento AGRARIA, località Feo di Vito snc, 89121 Reggio Calabria, Italy; 5 Dipartimento di Scienze Agrarie, Alimentari e Forestali, viale delle Scienze 11, 90128 Palermo, Italy

**Correction to: BMC Plant Biol 19, 7 (2019)**

**https://doi.org/10.1186/s12870-018-1576-y**

Following publication of the original article [1], the authors identified an error in Fig. 4. The correct figure is given below.

**Fig. 4:**
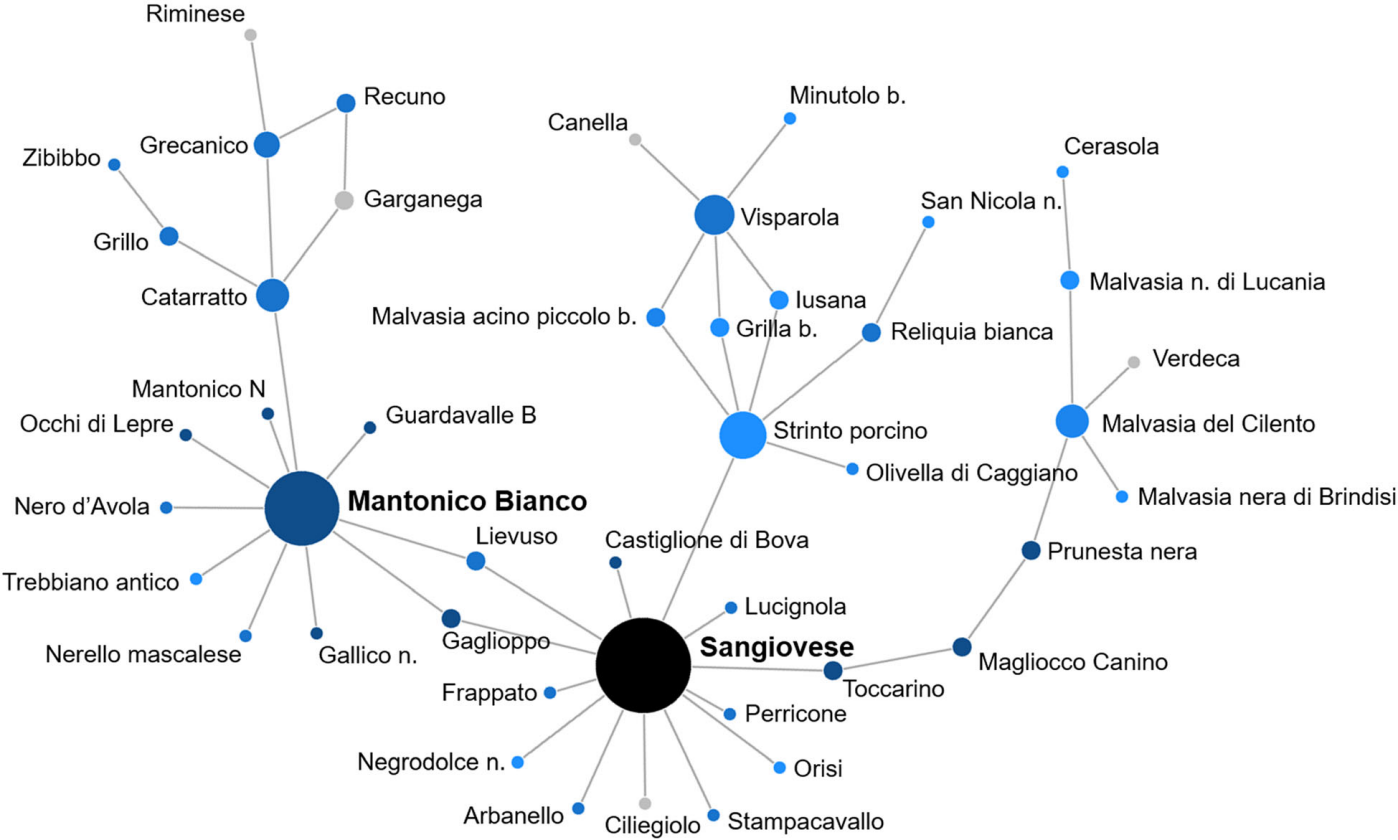
Network of first-degree relationships of Mantonico Bianco and Sangiovese. Vertices were colored based on the geographical origin of genotypes (Italy: blue scale based on sub-populations; genotypes from Laucou et al. [22]: grey; Reference: black) and their size was scaled based on the number of first-degree relationships of each genotype
