# Supplementary figures and images for: SNP genotyping elucidates the genetic diversity of Magna Graecia grapevine germplasm and its historical origin and dissemination
Source: BMC Plant Biol. 2019 Jan 6;19:7. doi: 10.1186/s12870-018-1576-y (PMC6322315; doi:10.1186/s12870-018-1576-y)

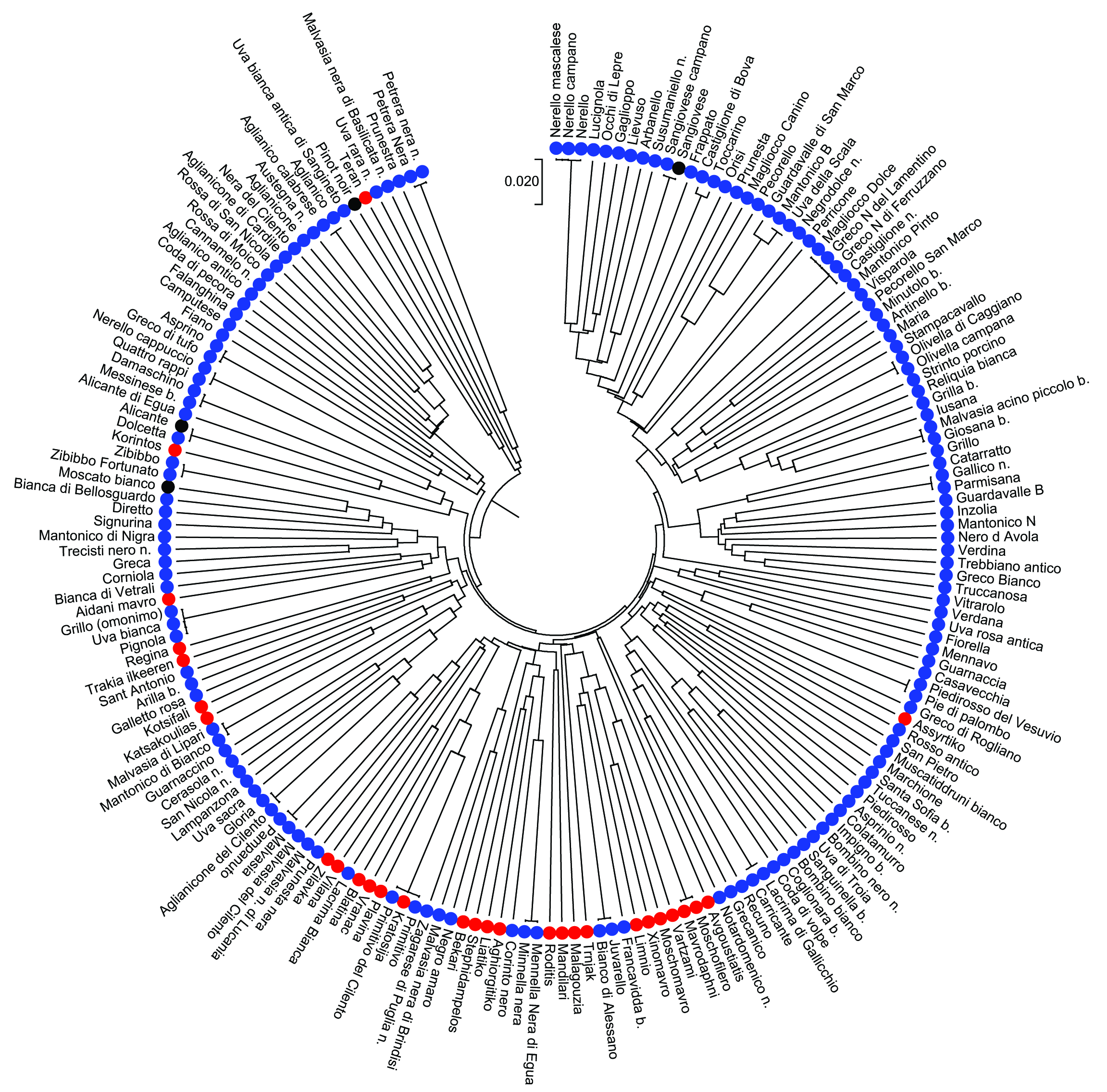

Supplement: Supplementary file 3 — UPGMA dendrogram of 187 grapevine genotypes analyzed by 18 K SNP array. The samples were marked based on their geographical origin. South Italy: blue dots; Eastern Mediterranean Sea: red dots; Reference: black dots. (TIFF 1630 kb) [file 12870_2018_1576_MOESM3_ESM.tiff]

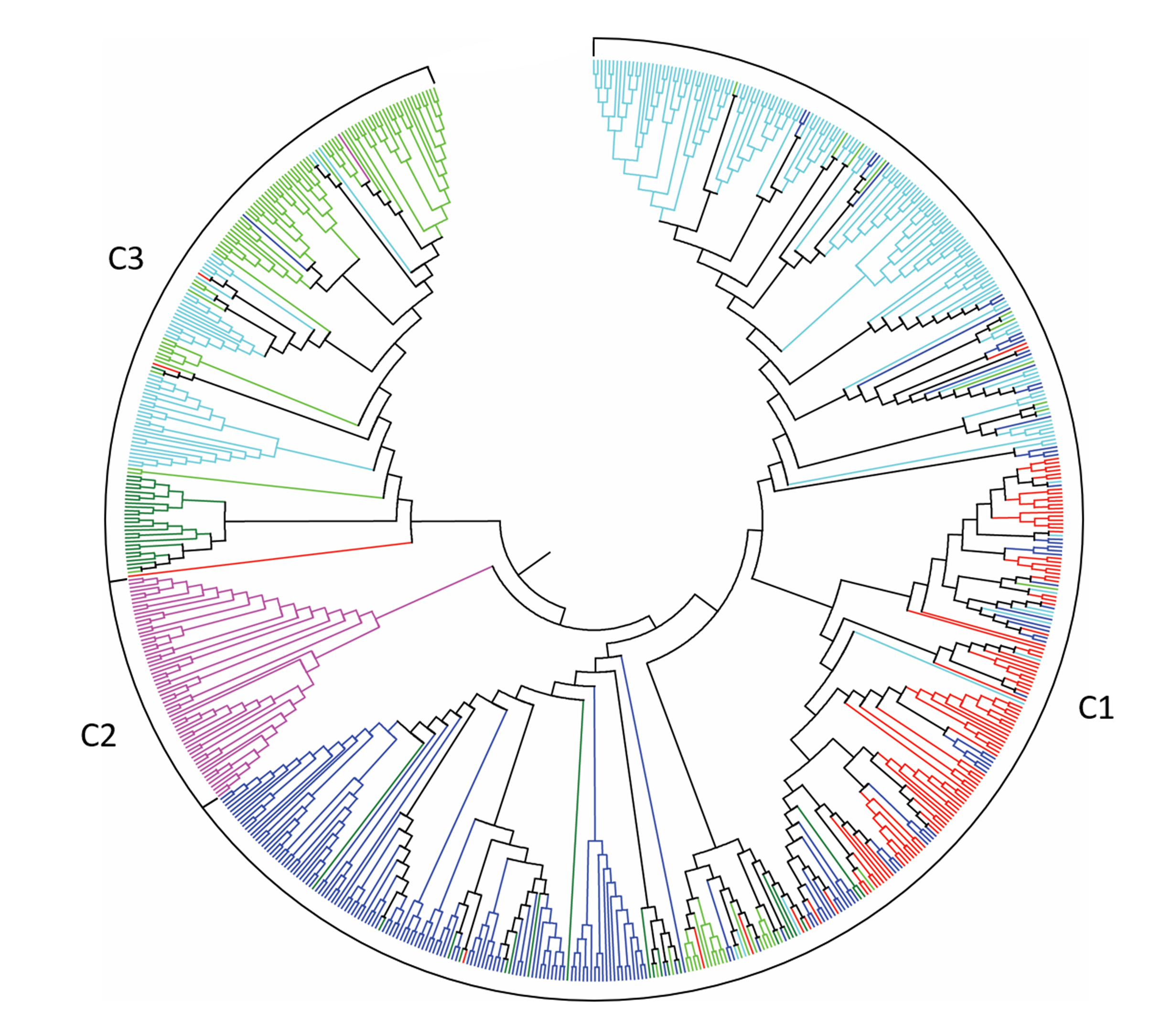

Supplement: Supplementary file 5 — Topology of UPGMA dendrogram of 709 grapevine accessions genotyped with 18 K SNP array. Georgia: violet branch lines; Eastern Mediterranean Sea: red branch lines; South Italy: blue branch lines; North Italy: dark green branch lines; France: light green branch lines; Iberian Peninsula: cyan branch lines. C1, C2, C3: main clusters identified. (TIF 2662 kb) [file 12870_2018_1576_MOESM5_ESM.tif]

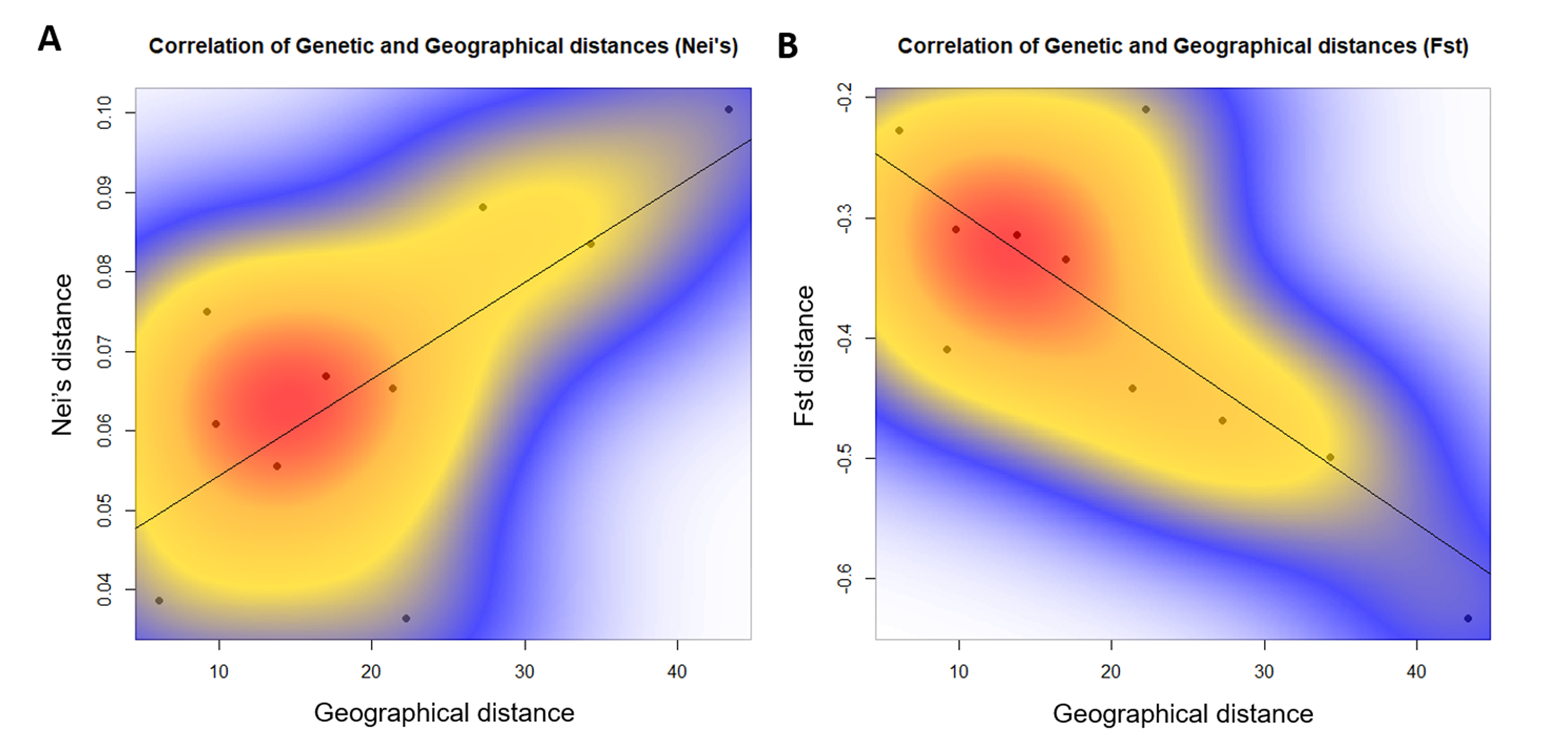

Supplement: Supplementary file 9 — Graphical representation of correlations between genetic distances and geographical distance. A: Nei’s genetic distance; B: Fst genetic distance. Red: high correlation between genetic and geographical distances; Yellow: medium correlation between genetic and geographical distances; Blue: low correlation between genetic and geographical distances. Dots represent distance values between two populations detected by DAPC. (TIFF 1753 kb) [file 12870_2018_1576_MOESM9_ESM.tiff]

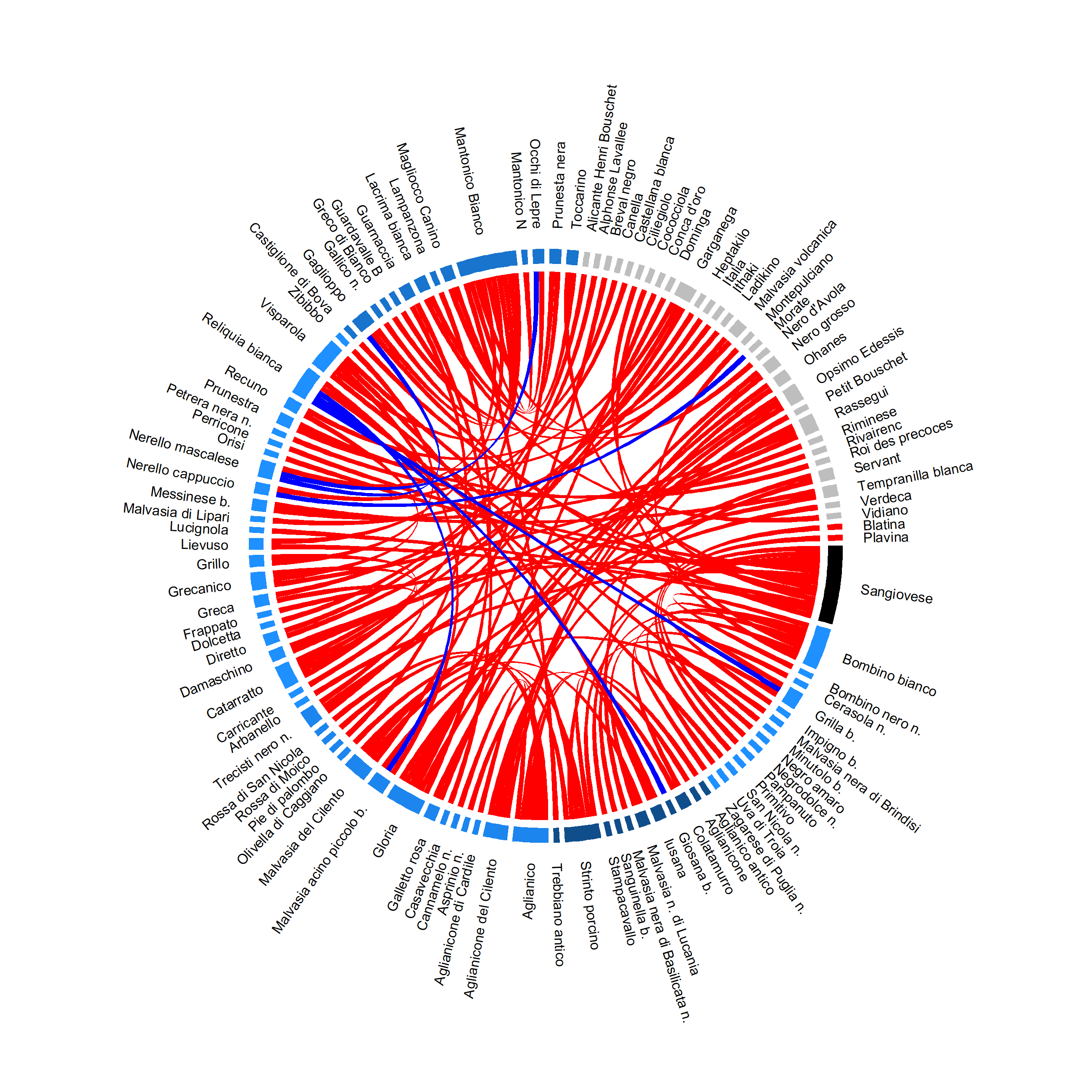

Supplement: Supplementary file 12 — Circular representation of first (red links) degree relationships identified for South Italy cultivars genotyped at 18 K SNP loci. The samples are arranged based on their geographic origin. Italy: blue scale (based on sub-populations); Eastern Mediterranean Sea Countries: red; genotypes from Laucou et al. [22]: grey; Reference: black. (TIFF 19774 kb) [file 12870_2018_1576_MOESM12_ESM.tiff]
