# Supplementary material for: SNP genotyping elucidates the genetic diversity of Magna Graecia grapevine germplasm and its historical origin and dissemination
Source: BMC Plant Biol. 2019 Jan 6;19:7. doi: 10.1186/s12870-018-1576-y (PMC6322315; doi:10.1186/s12870-018-1576-y)
Supplement: Supplementary file 4 — List of synonymies identified among the Magna Graecia germplasm (sample set #1) analyzed by 18 K SNP array. (DOCX 13 kb) [file 12870_2018_1576_MOESM4_ESM.docx]

**Additional file 4 -** List of synonymies identified among the *Magna Graecia* germplasm (set of samples #1) analyzed by 18K SNP array.

| **Synonymies** | **Comments** |
| --- | --- |
| Aglianico and Aglianichello | Confirmation of prior publication: [9] |
| Aglianicone del Cilento and Gloria |  |
| Alicante and Dolcetta | Confirmation of prior publication: [10] |
| Asprino and Greco di Tufo | Confirmation of prior publication: [9] |
| Bianco di Alessano and Iuvarello | Confirmation of prior publication: [15] |
| Carricante and Lacrima di Gallicchio |  |
| Castiglione n., Greco N di Ferruzzano, Greco nero del Lamentino and Magliocco dolce |  |
| Damaschino and Messinese b. |  |
| Gallico n. and Parmisana | Confirmation of prior publication: [65] |
| Giosana b. and Malvasia acino piccolo b. |  |
| Greco di Bianco and Malvasia di Lipari | Confirmation of prior publication: [15, 66] |
| Grillo (omonimo), Pignola and Uva bianca |  |
| Malvasia and Pampanuto |  |
| Mennella nera di Egua and Minnella nera | Confirmation of prior publication: [15] |
| Moscatello di Gallicchio and Petrera nera n. | Confirmation of prior publication: [15] |
| Nerello calabrese, Nerello campano and Nerello mascalese |  |
| Nerello cappuccio and Quattro rappi |  |
| Olivella campana and Olivella di Caggiano |  |
| Primitivo and Primitivo del Cilento |  |
| Sangiovese and Sangiovese campano |  |
| Zibibbo and Zibibbo fortunato |  |
