# Supplementary material for: SNP genotyping elucidates the genetic diversity of Magna Graecia grapevine germplasm and its historical origin and dissemination
Source: BMC Plant Biol. 2019 Jan 6;19:7. doi: 10.1186/s12870-018-1576-y (PMC6322315; doi:10.1186/s12870-018-1576-y)
Supplement: Supplementary file 8 — Nei’s standard genetic distance (below the diagonal) and Fst index (above the diagonal) calculated on five clusters inferred by DAPC. (DOCX 12 kb) [file 12870_2018_1576_MOESM8_ESM.docx]

**Additional file 8 -** Nei’s standard genetic distance (below the diagonal) and Fst index (above the diagonal) calculated on five clusters inferred by DAPC.

|  | Cluster 1 | Cluster 2 | Cluster 3 | Cluster 4 | Cluster 5 |
| --- | --- | --- | --- | --- | --- |
| Cluster 1 | - | -0.314 | -0.498 | -0.409 | -0.309 |
| Cluster 2 | 0.055 | - | -0.442 | -0.209 | -0.227 |
| Cluster 3 | 0.083 | 0.065 | - | -0.633 | -0.468 |
| Cluster 4 | 0.074 | 0.036 | 0.100 | - | -0.334 |
| Cluster 5 | 0.060 | 0.038 | 0.088 | 0.066 | - |
