# Supplementary material for: SNP genotyping elucidates the genetic diversity of Magna Graecia grapevine germplasm and its historical origin and dissemination
Source: BMC Plant Biol. 2019 Jan 6;19:7. doi: 10.1186/s12870-018-1576-y (PMC6322315; doi:10.1186/s12870-018-1576-y)
Supplement: Supplementary file 10 — Summary of four-population tests on five clusters inferred by DAPC. (DOCX 13 kb) [file 12870_2018_1576_MOESM10_ESM.docx]

**Additional file 10 -** Summary of four‐population tests on five clusters inferred by DAPC.

| **Populations used** | **ƒ4 statistic** | **SE** | **Z-score** |
| --- | --- | --- | --- |
| Cluster 1,Cluster 2;Cluster 3,Cluster 4 | 0.0035 | 0.0009 | 3.7011 |
| Cluster 1,Cluster 3;Cluster 2,Cluster 4 | -0.0027 | 0.0008 | -3.0504 |
| Cluster 1,Cluster 4;Cluster 2,Cluster 3 | -0.0062 | 0.0006 | -9.2147 |
| Cluster 1,Cluster 2;Cluster 3,Cluster 5 | 0.0015 | 0.0010 | 1.4910 |
| Cluster 1,Cluster 3;Cluster 2,Cluster 5 | -0.0031 | 0.0010 | -3.1540 |
| Cluster 1,Cluster 5;Cluster 2,Cluster 3 | -0.0047 | 0.0006 | -7.2797 |
| Cluster 1,Cluster 2;Cluster 4,Cluster 5 | -0.0019 | 0.0010 | -1.8932 |
| Cluster 1,Cluster 4;Cluster 2,Cluster 5 | -0.0040 | 0.0009 | -4.1449 |
| Cluster 1,Cluster 5;Cluster 2,Cluster 4 | -0.0020 | 0.0006 | -3.0700 |
| Cluster 1,Cluster 3;Cluster 4,Cluster 5 | -0.0004 | 0.0013 | -0.3240 |
| Cluster 1,Cluster 4;Cluster 3,Cluster 5 | 0.0022 | 0.0012 | 1.8229 |
| Cluster 1,Cluster 5;Cluster 3,Cluster 4 | 0.0026 | 0.0010 | 2.6041 |
| Cluster 2,Cluster 3;Cluster 4,Cluster 5 | 0.0015 | 0.0007 | 2.0313 |
| Cluster 2,Cluster 4;Cluster 3,Cluster 5 | 0.0007 | 0.0008 | 0.8323 |
| Cluster 2,Cluster 5;Cluster 3,Cluster 4 | -0.0008 | 0.0008 | -1.0248 |
